# Supplementary material for: Learning curve and surgical outcome of robotic assisted colorectal surgery with ERAS program
Source: Sci Rep. 2022 Nov 29;12:20566. doi: 10.1038/s41598-022-24665-w (PMC9709162; doi:10.1038/s41598-022-24665-w)
Supplement: Supplementary file 1 — Supplementary Information 1. [file 41598_2022_24665_MOESM1_ESM.zip › LSC Raw data-Ñ[▒K/106/H_ñw╝╨░O▒Kñσ.pdf]

正式摘要(電子簽章：0000002046231678)

W52 / W52 ☐英文標題(注意:無英文姓名-請補病人基本檔!) ☐【讀取】憑證

臺中榮民總醫院

出院病歷摘要

[redacted]

(8)流水編號	
(9)入院日期	109/11/26 (2020) 直腸外科 W52 - 143
(10)轉科日期	
(11)出院日期	109/12/03 (2020) 住院天數計 7 日

診斷

- 入院:sigmoid colon cancer
  hypertension
- 出院:1.sigmoid colon cancer s/p laparoscopic anterior resection on 2020/11/27
  2.hypertension

主訴

- bloody stool since 2020/09

病史

- This is a 55 y/o woman with history of HTN, who presented with bloody stool since 202009. She first went to 803 H. where colonoscope revealed rectal cancer. She then went to our CRS OPD where colonoscope revaled S colon tumor, FAV 20cm and ABD CT revealed S colon tumor. This time, she is admitte for operation.

體檢發現

- EGD: GRED +++ IN CSUH
  CAG
  [procedure] Reason For Test: HTN. ; Dizziness. Protocol: Bruce DURATION: 6:42 Exercise Tolerance: Good STAGE: III Chest Pain: Chest Pain: N Reason For Termination: SOB. ＆ Dizziness.＆ Recovery 0`20" give chair resting. Medication: □ Beta-block Bete-blockade Bete-blockade Bete-blockade Bete-blockade □ Calcium antagonist Calcium antagonist Calcium antagonist Calcium antagonist Calcium antagonist Calcium antagonist □ Coronary dilator Coronary dilator Coronary dilator Coronary dilator BP RESTING: 112 / 71 mmHg BP MAXIMUM: 172 / 77 mmHg MAX.MET: 8.90 HR RESTING: 85 bpm HR MAXIMUM: 146 bpm THR: 87 % Resting EKG: NSR. ARRHYTHMIAS: Nil. [Record] STAGE SBP DBP DBP HEART HR*SBP STAGE SBP DBP HEART HR*SBP (KMPH-GRADE) RATE (KMPH-GRADE) RATE REST 112 71 85 9520 0 Recovery 172 77 146 25112 I(2.7-10%) 112 71 125 14000 1 min REC 155 77 130 20150 II(4.0-12%) 151 72 141 21291 2 min REC 157 73 114 17898 III(5.4-14%) 172 77 146 25112 3 min REC 146 71 108 15768 IV(6.7-16%) 0 4 min REC 141 69 103 14523 V(8-18%) 0 5 min REC 129 68 103 13287 VI(8.8-20%) 0 6 min REC 121 67 101 12221 [ST CHANGES] LEAD ST mm REMARK RE
  - General appearance :
    - easy looking , conscious clear , without cardiopulmonary distress
    - Vital signs自動帶入date 體溫(BT) 脈搏(P) 呼吸(R) 血壓(BP) 身高(BH) 體重(BW) BMI
      20201126 36.7℃ 71次/分 18次/分 111/79mmHg 151.8cm 66.6kg 28.9
  - HEENT :
    - Eyes: no anemic conjunctiva, no icteric sclera, normal EOM
    - ENT: no otorrhea, no rhinorrhea, no erythematous mucosa, no injected tonsil, no deformity
  - Neck: supple, freely movable, trachea midline,no deformity, no engorged jugular vein , no palpable thyroid or lymph node enlargement
  - Chest:
    - Inspection: Grossly normal, symmetric and fully expansion, no accessory muscle used, no operation scar, no spider angiomata, no gynecomastia
    - Palpation: no palpable mass or nodule, no subcutaneous emphysema, no tenderness
    - Ausculation: clear breathing sound
  - Heart:
    - Percussion: normal heart size
    - Ausculation: regular heart beat, normal S1, S2, no gallop, no murmur
  - Abdomen:
    - Inspection: in
      - flat contour, symetrically, no scar, no stria, no dilated veins, no rashes
    - Ausculation: normoactive bowel sound, no abdominal bruits
    - palpation: soft, no muscle guarding or rigidity, no local tenderness, no rebounding pain, no palpable mass, no palpable pulsation
    - Percussion: no shifting dulllness, no hepatomegaly, no splenomagaly
  -

Peripheral pulsation :	CA	BA	RA	FA	PA	PTA	DPA
Right side :	++	++	++	++	++	++	++/+++
Left side :	++	++	++	++	++	++	++/+++

  - Genito-anus: no tenderness or palpable mass lesion or rectal shelf, normal external genitalia, normal anal tone, brown stool

CRS CONDITION

  -
    - Lung

手術日期及方法

- 手術日期及方法20201127 1. Intraoperative colonoscopy : (-)
  2. High ligation (+) : At IMA root
  3. The colon was not taken down from the splenic flexure
  4. EndoGIA (60-3.5) X1 + EEA (29mm) were used
  5. Cut margin: Proximal 8 cm, distal about 4 cm
  6. No drainage tube was inserted
  7. The ADHESION BARRIER FILM was placed below the umbilicus extended wound.

住院治療經過

- After admission, operation with laparoscopic anterior resection was done on 11/27 as scheduled. We let her try water on POD1 and liquid diet on POD3 as her tolerance and then increased feeding amount. Under the stable condition, she was discharged on 12/3.

合併症

- nil

一般檢查

- 累積報告：

DATE	TIME	ALB	TP	BIL.T	BIL.D	ALKP	AST	ALT	LDH	CHOL	TG	HDL-C	LDL	UA	NH3	AMY
1091126	1838	4.4	7.1	0.44	0.10	61	48	75	193							
1091127	1634	4.0		0.4				68								
1091130	0943			0.4				22								
1091203	0935						15	13								

DATE	TIME	WBC	RBC	HGB	HCT	MCV	PLT	BLST	NEUT	BAND	SEG	LYM	MON	EOS	BAS
1091126	1710	6250	4.60	12.0	37.2	80.9	398		69.7			22.9	4.0	2.9	0.5
1091127	1658	12750		10.8	33.5		330		87.0			10.0	2.6	0.2	0.2
1091130	0932	6360		10.7	32.4		332		72.1			20.0	4.4	3.0	0.5
1091203	0922	5890		10.3	30.6		333		74.4			16.3	4.6	4.4	0.3

DATE	TIME	NA	K	CL	CA	BUN	CREAT	Mg	P	CRP	ESR	PCT	LIPASE	LACTATE
1091126	1838	141	3.4	103	9.2	10	0.77							
1091127	1634	143	3.4		8.3	11	0.70							
1091130	0943	139	3.5	105	8.3	7	0.63							
1091203	0935	140	3.5		8.1	12	0.62							

DATE	TIME	GLUCOSE	PAB	TRF	ZN	CU	KETONE	CK	CKMB	PT-P	PT-C	APTT-P	APTT-C	TRO-T
1091126	1710									10.6	10.3	26.6		
1091126	1712													5.47
1091126	1713								4					
1091126	1838							124						

- 放射線報告：檢驗項目 **:CT ABDOMEN-SURVEY** 報告日期 **:1091125**

  主　訴：

  Result:

  Finding:

  1. Imaging Modality and protocol:
     CT of abdomen with IV and oral contrast.
  2. Tumor location and size:
     R/O rectal Ca; Mild circumferential wall thickening of rectum; the tumor location and size are difficult to be determined.
  3. Tumor invasion:
     Not accessible
  4. Regional lymph node metastasis:
     Not accessible
  5. Distant metastasis:
     No evidence of abnormal lymphadenopathy in paraaoratic space.
  6. Other findings:

  > fatty infiltration of liver
  > small mesentery LNs over right lower abdomen,
  > Engorgement of left adnexal and gonadal vessels, r/o pelvic congestion syndrome.
  > Presence of a soft tissue mass arising from left adnexa, 23 mm in diameter, with cystic component, in favor of ovary/cyst; please GYN clinical correlation.

  Impression:
  > R/O rectal Ca, for staging; For details, please see the descriptions above.

特殊檢查

病理報告

其它

出院時情況

- 3.改門診治療

出院指示

藥名	劑量	劑量單位	用法	頻次	數量	數量單位	現況	天數	單位
$Biofermin tab	2	TAB	PO	TID	42	TAB	TH	7	天
Acetylcysteine tab	1	TAB	PO	QN	7	TAB	TH	7	天

出院計劃

主治醫師 2062F 蔣鋒帆 / 住院醫師 2331F 陳凱祥

正式摘要(電子簽章：000000204621678)

W52 / W52

doctorRank:B
